# The Effect of an Educational Intervention on the Knowledge and Self-Care Skills of Adult Patients With Venous Leg Ulcers: Protocol for a Randomized Controlled Trial

**DOI:** 10.2196/84211

**Published:** 2026-04-21

**Authors:** Cristina Canova, Jessica Scappin, Maria Vittoria Chiaruttini, Laura Tasson, Flavia Masiero, Claudia Varagnolo, Matteo Martinato

**Affiliations:** 1 Department of Cardiac Thoracic Vascular Sciences and Public Health, Unit of Biostatistics, Epidemiology and Public Health, University of Padova, Via Loredan, 18, Padova, 35131, Italy, 39 3474185474; 2 Azienda ULSS 6 Euganea, Padova, Italy

**Keywords:** education, venous leg ulcer, randomized controlled trial, knowledge, self-care skills

## Abstract

**Background:**

Venous ulcers have a prevalence of 1% in Western countries, with a tendency to become chronic and recur frequently. The lengthy periods of healing required for the treatment of these lesions result in increased costs. It is possible that patient education may enhance patients’ understanding of their health condition, which could potentially lead to a reduction in recurrence rates. Nevertheless, it is uncertain whether this constitutes the optimal educational strategy.

**Objective:**

The objective of this study is to assess the superiority of an educational intervention delivered via video compared to an informative brochure in outpatient nursing clinics, focusing on patients’ knowledge of venous leg ulcers and their self-care skills.

**Methods:**

A total of 144 patients with venous leg ulcers who attend the outpatient nursing clinics will be recruited to participate in this randomized controlled trial. Individuals with cognitive impairment, an ankle-brachial index of less than 0.90, arterial insufficiency, and arterial ulcers or ulcers of mixed origin will be excluded from the study. The intervention comprises 4 short educational videos specifically created for this study, in which patients will be provided with information on disease knowledge, medication management, compression therapy, and the importance of adequate nutrition and physical activity. The control group will be provided with the standard education offered at the outpatient clinics participating in the study, which will be disseminated through an information leaflet. Patient assessments will be performed with standardized, validated tools at baseline; at 1, 3, and 6 months postenrollment; and at 12 months via telephone follow-up. The primary outcomes are the patient’s level of knowledge and their ability to self-care. The secondary outcomes are the patient’s level of well-being, the success rate of the treatment, and the recurrence rates. A preliminary feasibility study was conducted to methodologically refine the protocol.

**Results:**

A feasibility study was conducted to refine study procedures and data collection tools, confirming the acceptability of the questionnaires and the feasibility of digital data collection. The randomized controlled trial was funded in June 2025. It is currently pending ethics approval, and it is expected to start recruitment in spring 2026. Results from the main trial are expected to be available by the end of 2027.

**Conclusions:**

A video-based educational intervention may prove to be an effective, readily accessible, and replicable method of patient education. The findings of this study will inform the most effective method of patient education for this population, ensuring the delivery of high-quality care and enhancing the quality of services.

## Introduction

Venous leg ulcers (VLUs) are defined as skin lesions on the lower leg or foot with a healing time of more than 6 weeks [1]. These dermatological conditions occur in the lower extremities as a consequence of elevated venous pressure, leading to vascular dilation and fluid stagnation in the affected areas. VLUs are a complication of chronic venous insufficiency, which causes damage to the vessel walls and surrounding tissues. They account for 70% of all leg ulcers [1]. In addition to venous insufficiency, several factors, such as age, reduced mobility, and obesity, have been identified as contributing to the development of VLUs [2].

The incidence of VLUs is increasing among adults, particularly among older adults who often have concomitant diseases. It affects 1% of the global population between the ages of 18 and 64 [3]. In the United States and other Western countries, the prevalence of this condition is estimated to be approximately 3% among the older adults [4-6].

In clinical practice, the treatment of VLUs typically involves prolonged healing periods and a high rate of recurrences [7,8]. A review of the literature shows that 22% of individuals who recover from VLUs will experience a recurrence within 3 months, 57% within 1 year, and up to 78% within 3 years. Furthermore, the risk of recurrence is 4.4 times higher in patients with a history of multiple VLUs [1], leading to increased treatment needs and health care costs. The diagnosis, treatment, and cure of VLUs account for up to 2% of the annual health care budget of Western societies [6].

A wound that persists for more than 1 month without following the normal healing process is classified as a chronic wound [3]. This chronicity is associated with a diminished quality of life. Patients frequently report a range of symptoms and experiences, including pain, sleep disturbances, malodor, impaired mobility, altered body image, reduced energy levels, and dissatisfaction with treatment. These symptoms and experiences can lead to a significant decline in perceived quality of life, encompassing physical, psychological, and social domains [1,9,10]. Additionally, VLUs can result in leg weakness, swelling, exudation, and recurrent infections [4].

Given the chronic and prevalent nature of these ulcers, patients or their caregivers frequently take on the responsibility of managing and providing self-care for VLUs. This involves maintaining health, monitoring the condition, and managing any issues that arise. The maintenance phase focuses on the prevention of injuries or recurrences through the reduction of risk factors. Monitoring involves observing signs and symptoms, while management encompasses the patient’s response to any observed signs and symptoms [8]. A significant barrier to effective VLU self-management is the lack of adequate health information [11]. This gap in knowledge limits the ability of patients or their caregivers to maintain health, monitor the condition, and manage the wound effectively. Therefore, it is crucial to ensure that patients are fully informed about their health condition, associated risk factors, potential complications, protective measures, and available treatment options for managing symptoms.

Noninvasive treatments for VLUs include the application of local dressings to the wound and surrounding skin, as well as the use of compression therapy. The literature shows that compression therapy is a more effective method for promoting healing compared to the application of local dressings alone, resulting in a reduction of the wound surface area by 30% [4]. Furthermore, the proper application of compression therapy has been demonstrated to reduce pain, alleviate pressure, and decrease volume overload in the venous system [12], thereby improving venous and lymphatic return [4]. Given the high recurrence rates at 12 months postwound healing, it is crucial to maintain preventive measures, particularly compression therapy, in order to mitigate the risk of recurrence [13,14].

Unfortunately, a significant proportion of patients either do not adhere to or only partially adhere to compression treatment [15]. Nonadherence to recommended compression therapy is associated with a significant increase in the risk of delayed wound healing and recurrence, with estimates ranging from 2 to 20 times higher [5]. Adherence is influenced by a number of factors, including receiving conflicting information [16] or a lack of clear, objective, and easily understandable information [17]. Additionally, adherence is influenced by the individual’s beliefs about the effectiveness of the treatment, their awareness and knowledge of the condition [14], and the level of patient involvement in the management of their VLU [3]. A deeper understanding of their condition empowers patients to engage in effective self-care and avoid risky behaviors [8]. Therefore, it is crucial to promote adherence to continuous compression therapy through patient education [17] to enhance knowledge and prevent recurrence.

However, the consistency of patient education across different health care settings remains variable, and there is a lack of robust scientific evidence regarding the effectiveness of interventions [14,18]. The educational interventions provided to patients with VLUs in outpatient clinics often lack clear and consistent guidelines for delivery, with the approach being dependent on the individual health care provider. In cases of high workload, a standardized tool is not always guaranteed, and education may be completely absent, as reported by the clinicians involved in this care setting.

A method for patient education described in the literature involves face-to-face sessions lasting 20 to 60 minutes, conducted at the patient’s home or in the outpatient clinic, with or without written material [1]. However, given the high prevalence of VLU, it is often not feasible to carry out such lengthy sessions due to staffing limitations. Another effective educational method involves participation in a community of VLU or healed patients, where experienced VLU nurses provide education. However, transportation issues and limited availability make it difficult for all patients to attend these meetings [19].

Another potential strategy for the dissemination of information and the promotion of awareness is the use of manuals, brochures, or small books. These resources can facilitate the dissemination of knowledge and enhance the understanding of the general population, enabling patients and their families to refer back to them multiple times and support decision-making in the management of VLU [20]. A study conducted in Italy at a vulnology clinic for ambulatory patients with VLUs demonstrated that providing an informational brochure can improve patient education, particularly in self-care capacity and disease knowledge [15]. Conversely, a study conducted by Baquerizo Nole et al [21] suggests that illustrative video recordings may be even more effective in enhancing patient knowledge compared to brochure-based education.

Indeed, educational videos are easily accessible, can be displayed in clinic waiting rooms, do not require the ability to read or comprehend written text, and also ensure that patients receive accurate and consistent information, reducing communication errors attributable to the disparate experience of health care providers or potential information distortion. In addition, video-based education could facilitate personalized care by allowing patients to request further information or specific advice from professionals. This enables nurses to prioritize delivering more relevant guidance instead of repeating information the patient may already know. These considerations make them a potentially superior method of education. However, the results of previous studies have not been statistically significant due to the limited number of enrolled patients [21].

On these premises, a project was developed to assess the superiority of an educational intervention delivered via video compared to an informational brochure in outpatient nursing clinics, focusing on patients’ knowledge of VLUs and their self-care skills. The aim of this article is to present the protocol for this clinical trial.

## Methods

### Study Design

#### Study Setting and Design

The study is a randomized, controlled, single-blind trial with 2 parallel groups. The aim is to assess the effectiveness of the experimental intervention in increasing the level of knowledge and self-care skills of patients with VLUs compared to the standard of care provided in outpatient nursing clinics, where education is delivered through an informational brochure. The intervention comprises an educational video on VLU management. Patients are randomized using block randomization in order to prevent imbalance between the intervention and control groups over the course of the 12-month study period.

As a parallel project, a longitudinal prospective substudy will be dedicated to validating the Italian version of the Well-being of Wounds Inventory (WOW-I) questionnaire.

The protocol was developed in accordance with the SPIRIT (Standard Protocol Items: Recommendations for Interventional Trials) 2013 statement [22].

#### Eligibility Criteria and Exclusions

Patients attending the outpatient nursing clinics involved in the study will be enrolled. To be eligible to participate in the study, patients must meet all of the criteria set out in Textbox 1. These include being aged 18 years or older, having a VLU, understanding Italian, being able to read and comprehend the questions, providing consent to participate in the study, and having access to a smart device with internet connectivity or a caregiver able to provide such a device. Individuals with cognitive impairment, an ankle-brachial index of less than 0.90, arterial insufficiency, or ulcers of arterial or mixed origin will be excluded from participation.

Textbox 1. **Eligibility criteria.**

**Inclusion criteria**

- Aged 18 years or older
- Affected by venous leg ulcer
- Able to understand the Italian language
- Able to read and understand the questions
- Attending the nursing clinics involved in the study
- Provided consent to participate in the study
- Able to use a smart device with internet access or supported by a caregiver able to use a smart device as a video player

**Exclusion criteria**

- Cognitive impairment
- Ankle-brachial index less than 0.90 or arterial insufficiency
- Arterial or mixed-origin ulcers

#### Randomization and Blinding

After obtaining informed consent and confirming eligibility criteria, randomization will be conducted using the REDCap (Research Electronic Data Capture; Vanderbilt University) platform. Patients will be randomly assigned to 1 of 2 parallel groups in a 1:1 ratio, using block randomization to ensure balance between the 2 groups during the follow-up.

The study is single-blind. Two separate teams, blind and unblind staff, will be created and assigned different activities to be performed. Nurses belonging to the blind staff will be responsible for assessing patients and delivering a blinded QR code, thereby preventing them from knowing which intervention is assigned to each participant. The unblind staff will have access to all other information except clinical assessments and will serve as the appointed contact point for patients and their caregivers regarding any study participation–related issues, thus preventing any potential risk of unblinding. Patients’ and caregivers’ questions (related to study participation or clinical care) will be directed only to the unblind staff. Both the blind staff and the patients or caregivers will be informed of this logistic before the study starts and reinforced during the study conduction and follow-up.

#### Experimental Treatment

The experimental educational intervention consists of 4 short, professionally produced videos covering the following topics: VLU knowledge, medication management, compression therapy, and diet and physical activity.

The content of the videos was derived from a thorough literature review and further refined by 2 expert nurses specializing in wound care. The videos were recorded by an experienced videographer in collaboration with a patient volunteer, 2 nursing students, and 2 nurses with expertise in managing VLUs.

In each video, the presenter provides clear and accessible explanations, using language that is easily comprehensible to the general public. When medical terminology is used, a definition is provided. Additionally, key terminology used by the speaker is displayed on the screen, accompanied by realistic images and scenes relevant to the discussed topics, enhancing comprehension through visual representation.

The first video is approximately three and a half minutes long and provides an overview of VLUs, their anatomical location, the associated risk factors, the mechanisms of development, and the diagnostic criteria.

The second video, lasting approximately 5 minutes, provides an in-depth exploration of the importance of treating VLUs, various management and treatment approaches, and potential complications.

The third video, 7 minutes long, explains the concept of compression therapy, its benefits and contraindications, the proper technique for applying elastic stockings, and lifestyle changes, including advice on physical activity to prevent the development of ulcers.

The fourth video, which lasts about 2 minutes, presents a list of foods and nutrients that promote the healing of VLUs and help prevent their recurrence.

The videos were uploaded to a YouTube channel accessible only to individuals possessing the requisite link or QR code. This was done to prevent contamination and ensure the integrity of the study. The platform allows the researcher to determine the number of views, which serves as an indicator of adherence to the experimental treatment. To track the views of each participant, every person in the experimental group will receive the same videos but will view a differently uploaded version.

Consequently, each video link or QR code will be associated with an alphanumeric identification number assigned to a participant, a strategy that will allow researchers to link the questionnaires completed by each participant to the number of views of the individual videos. Participants will be required to retain the link or QR code for viewing the intervention, as it will differ from the links or QR codes of other participants in the study.

#### Video Intervention

A document containing an identification number and QR codes or links will be provided to participants who have been assigned to the intervention group. The identification code, which the patient is required to retain and keep secret, is alphanumeric and random. The code is used by the participant to complete the questionnaires at baseline and during the follow-up periods, with the intention of guaranteeing anonymity. The QR codes and links serve the purpose of enabling the patient to access the 4 experimental educational videos. Patients assigned to this group will be required to access the videos via QR codes or links and view them after completing the baseline questionnaires or within the initial 7-day enrollment period. The educational intervention can be viewed in the outpatient nursing clinic after the visit, in a waiting room, with one’s own smart device or a device provided by the company, or at the participant’s home.

The videos may be viewed on multiple occasions during the study period. The outpatient nurse will issue a reminder to the patient to view the intervention at least once.

#### Control Treatment

Similarly, the control group will receive a document containing an alphanumeric identification number and a link or QR code. The identification code will be used in the same way as it is used for the intervention group, allowing participants to complete the questionnaires at the baseline and follow-up stages. The QR code will instead direct participants to a printable educational brochure. This brochure represents the standard treatment. It was introduced following a study conducted in 2021 that demonstrated its efficacy in increasing patient compliance, self-care skills, and disease knowledge in patients with VLUs [15]. Since then, it has been routinely used to educate VLU patients in the study settings. The educational content in the brochure covers some of the topics presented in the educational videos but in a short form, such as the nature of venous ulcers, the available treatments and compression therapy, dressing management, potential risks and complications, and beneficial lifestyle changes. The brochure will contain both written text and illustrative images and will be available to participants throughout the duration of the study.

#### Baseline Questionnaires

Patients who meet the eligibility criteria and consent to participate in the study will be requested by the researcher to provide sociodemographic and health-related data, including age, gender, level of education, occupation, with whom they live, presence of private or family care, preexisting pathologies, smoking habits, presence of pain, venous ulcer–related information, and medication management. The researcher will input these data and the Pressure Ulcer Scale for Healing (PUSH) Tool 3.0 measurement scale into the REDCap platform using a tablet provided by the company. To ascertain the level of knowledge about the disease, the level of self-care, and the patient’s well-being, the patient will be required to complete, respectively, the validated Venous Leg Ulcer Knowledge (VLUK) measurement scale, the validated Venous Leg Ulcer Self-Efficacy Tool (VeLUSET) measurement tool, and the WOW-I scale, using the company’s or their own smart device.

#### Follow-Up

Once the baseline questionnaires have been completed, patients will be invited to view the educational intervention assigned to them. Subsequently, at 4-week, 3-month, and 6-month intervals, participants will be required to complete the same questionnaires as at baseline. The VLUK, VeLUSET, and WOW-I scales have been employed to permit a comparison of pretreatment and posttreatment scores. Additionally, the outpatient nurse will be assigned to evaluate the patient’s venous ulcer using the PUSH Tool 3.0.

The final follow-up is scheduled to occur 12 months after enrollment and will be conducted via a telephone interview. Patients will be asked whether the condition has healed and, if so, for how long. Additionally, patients will be asked whether the ulcer has recurred.

#### Setting and Data Collection Method

The study will be conducted in outpatient clinics located in a northern Italian region.

The data will be collected by the nurses of the outpatient clinics involved in the study. Their duties will include checking eligibility criteria, collecting sociodemographic and health data at baseline, distributing the paper containing the unique code and QR codes for accessing the educational intervention and for completing the other questionnaires, and entering the data into the PUSH Tool 3.0 at baseline and at each follow-up. The remaining questionnaires (VLUK, VeLUSET, and WOW-I) are to be completed by the participants themselves at the outset of the study and at each subsequent follow-up. Data collection forms will be accessible through the electronic case report form. Data will be collected anonymously via the REDCap platform on a smartphone or tablet provided by the company or the patient.

#### Monitoring and Data Management

The researcher will be responsible for the collection of data at the time of the enrollment of the patient, either directly or through adequately trained personnel. Furthermore, the researcher will provide guidance to the nurse on the correct collection of data during the follow-up period, specifically in relation to ulcer assessment and the patient’s exit from the study.

It is not possible to exercise control over the questionnaires completed by patients themselves, as they are completed anonymously. During outpatient visits, the nurse will remind the patient or caregiver to view the educational intervention at least once by scanning the QR code and to complete the questionnaires during follow-ups.

The management, processing, and statistical analysis of the data will be conducted by external parties in collaboration with the sponsor. These parties will retain the data only for the duration necessary to complete the tasks assigned to them.

### Outcomes Measures

#### Primary Outcomes

The 2 coprimary outcomes of this study are an improvement in patient knowledge about their disease (VLUK score) and improvement in self-care skills (VeLUSET score) at 4 weeks.

#### Secondary Outcomes

Secondary outcomes (descriptive) include (1) VLUK and (2) VeLUSET scores at 3 and 6 months, (3) the evaluation of the healing process (PUSH Tool 3.0 score), and (4) improvements in well-being (WOW-I score) at 1, 3, and 6 months.

Moreover, the number of VLU recurrences in participants and the number of patients with complete healing will be assessed at 12 months of follow-up.

The instruments used to investigate the primary outcomes and well-being are self-administered questionnaires, while the healing process will be evaluated by the nurse using the PUSH Tool 3.0. The questionnaires and tools will be administered at the beginning of the study and at subsequent follow-up points, as described below in the study timeline.

**Figure 1.**
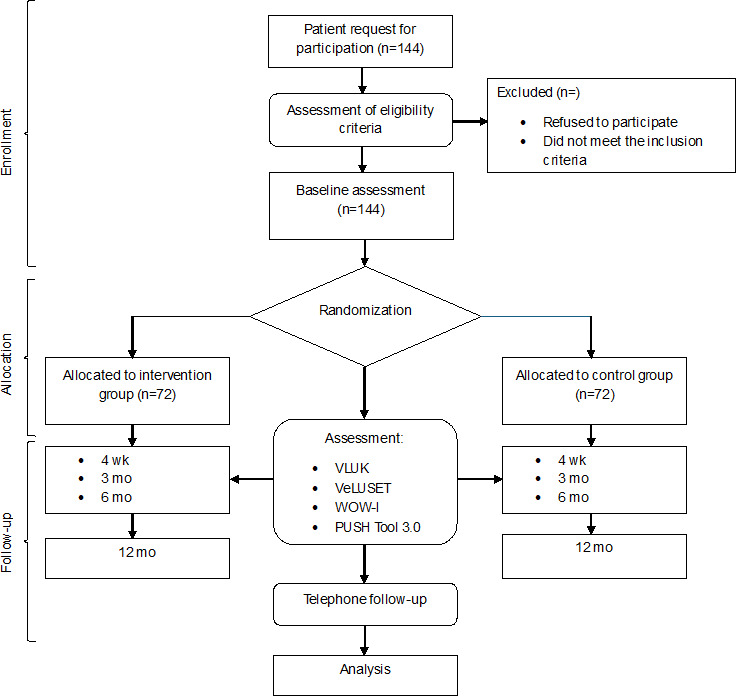
Study flowchart. PUSH Tool 3.0: Pressure Ulcer Scale for Healing Tool 3.0; VeLUSET: Venous Leg Ulcer Self-Efficacy Tool; VLUK: Venous Leg Ulcer Knowledge; WOW-I: Well-Being of Wounds Inventory questionnaire.

Finally, the presence of any recurrence and complete healing will be investigated at the 12-month follow-up point through a single question asked by the nurse via telephone. No specific tools will be employed. The previously described evaluations will be conducted in both the intervention and control groups (Figure 1).

#### Description of Questionnaires and Tools

##### Venous Leg Ulcer Knowledge

Patient knowledge about the disease will be assessed using the questionnaire developed for the study “Educational Interventions in Patients with Venous Leg Ulcer Patients” (VLUK) tool, a validated instrument in the Italian context [6]. The VLUK assessment tool comprises 15 multiple-choice questions, each with one correct answer, designed to evaluate the patient’s knowledge of VLU pathophysiology, treatments, and recommended lifestyle changes [15].

##### Venous Leg Ulcer Self-Efficacy Tool

Patients’ self-care skills will be assessed using the VeLUSET, which has been validated in the Italian context in another unpublished study [23]. This tool assesses 5 dimensions of self-care in people with VLUs. The tool comprises 30 items, which can be grouped into 5 areas: general self-care (5 items), daily self-care activities (12 items), normal living (4 items), skills development (6 items), and trauma avoidance (3 items) [16,24].

##### Well-Being of Wounds Inventory

Patients’ well-being will be evaluated using the WOW-I scale, which will be translated and validated in the Italian context.

This scale assesses the well-being of the patient with VLUs in relation to their overall quality of life, their social circumstances, and the specific experience of the wound. The scale comprises 2 subscales, the first of which includes 2 factors or categories and the second of which has 5 factors or categories, resulting in a total of 19 items, each evaluated using a Likert scale [25].

##### PUSH Tool 3.0

The healing process will be documented within the study using the PUSH Tool 3.0, introduced in 1997 by the National Pressure Ulcer Advisory Panel and demonstrated to be an effective method for monitoring the healing of venous ulcers [26]. The tool was selected for its simplicity, speed, and reliability in monitoring venous ulcer healing. It comprises 3 items: lesion size (length × width), exudate quantity (none, light, moderate, and heavy), and tissue type (necrotic tissue, slough, granulation tissue, epithelial tissue, and closed). The total lesion score is obtained by summing the single-item scores, which range from 0 to 17. A reduction in the total score is indicative of progress toward the healing process.

If a patient achieves complete healing before the conclusion of the study, they will be classified as a “healed patient” and contacted again via telephone for follow-up purposes.

### Validation of the Italian Version of the WOW-I Questionnaire

To psychometrically validate the Italian version of the WOW-I, patients will be invited to complete the WOW-I questionnaire, the “EuroQol-Visual Analog Scale” [27], and the well-being subscale of the “Cardiff Wound Impact Schedule” [2], at baseline and 1 week later. Internal consistency will be assessed using Cronbach α at both time points. Stability over time will be evaluated by mean differences in test-retest scores, particularly in relation to well-being levels assessed via the EuroQol-Visual Analog Scale. Convergent validity will be estimated by correlating WOW-I subscales with the Cardiff Wound Impact Schedule well-being subscale.

### Study Timeline

The study will be conducted over a period of 1 year from the end of enrollment. After enrollment (t_0_), the investigator will administer the WOW-I questionnaire to the patient at 7 ± 2 days (t_1_) to verify the stability of the instrument, and then 3 other follow-up assessments are planned for the primary outcomes. The second follow-up evaluation (t_2_) is scheduled to take place 4 weeks after the educational video is delivered, with subsequent follow-up assessments (t_3_) at 3-month intervals and 1 at 6 months (t_4_). If a patient is healed before the end of follow-up, they will be classified as a “healed patient.”

Finally, a long-term telephone follow-up will be conducted 12 months (t_5_) after enrollment (Table 1).

**Table 1.** Summary of time points, interventions, assessments, and data collected.

	Enrollment	Postallocation				Closeout
	t_0_	t_1_ (1 wk)	t_2_ (4 wk)	t_3_ (3 mo)	t_4_ (6 mo)	t_5_ (12 mo)
Enrollment						
Eligibility screen	✓					
Informed consent	✓					
Allocation	✓					
Interventions						
Intervention extra: video		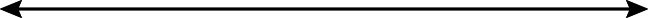				
Intervention standard: brochure		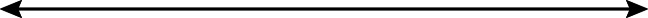				
Assessments						
Sociodemographic factors	✓					
Health factors	✓					
PUSH Tool 3.0^a^	✓		✓	✓	✓	
VLUK^b^	✓		✓	✓	✓	
VeLUSET^c^	✓		✓	✓	✓	
WOW-I^d^	✓	✓	✓	✓	✓	
Ask if there has been healing or if there is a recurrence						✓

a PUSH Tool 3.0: Pressure Ulcer Scale for Healing Tool 3.0.

b VLUK: Venous Leg Ulcer Knowledge.

c VeLUSET: Venous Leg Ulcer Self-Efficacy Tool.

d WOW-I: Well-being of Wounds Inventory questionnaire.

### Sample Size Calculation

The primary end points are the mean differences in pre-post scores (collected after 4 wk of follow-up and at baseline) of the VLUK and the VeLUSET questionnaires, which are considered coprimary hierarchical end points. This implies that the mean difference in VLUK within the treatment group must be statistically greater than the mean difference in the control group before the mean difference in VeLUSET between the control and treatment groups is evaluated. An interim analysis has been planned at the halfway point of the enrollment. The interim analysis assumes the Lan-DeMets O’Brien-Fleming approximation alpha-spending function and includes an early efficacy stop in case *Z*>2.54. The nominal *P* values are .006 and .048 at the interim and final analyses, respectively. Moreover, the conditional power will be calculated, and resampling will be performed in case of 50%<conditional power<80%. Otherwise, the sample size will be kept at the original size (72 patients per group), as described in the following. Particularly, a type I error of 5%, a power of 80%, and a dropout rate of 10% have been considered for the sample size estimation.

### Statistical Analysis

A descriptive analysis will be conducted to summarize the baseline characteristics of the 2 randomized groups. Continuous variables will be analyzed using means and standard deviations (assuming normality) or medians and interquartile ranges. Categorical variables will be analyzed using proportions and frequencies.

Primary analysis will be conducted according to the intention-to-treat principle. The 2 coprimary outcomes, change in VLUK from baseline and VeLUSET scores at 4 weeks, will be analyzed using generalized linear models with a Gaussian family and identity link, corresponding to an analysis of covariance framework. For each outcome, the post-intervention score at 4 weeks will be used as the dependent variable, with the treatment group as the main independent variable and the corresponding baseline score included as a covariate. In the event of an imbalance between the 2 groups, multivariate generalized linear models will be employed. The coprimary outcomes will be evaluated using a hierarchical testing strategy to control the family-wise type I error rate at 5%. Specifically, the between-group difference in VLUK will be tested first; only if this comparison reaches statistical significance will the coprimary outcomes be assessed at the same significance level (ie, 5%). The secondary outcomes assessed at follow-up will be analyzed, accounting for the repeated-measures structure of the data, with time treated as a categorical variable, using appropriate parametric methods for longitudinal data. When relevant, within- and between-group comparisons over time will be performed.

Comparisons of categorical outcomes, including healing status and recurrence, will be conducted using chi-square or Fisher exact tests, as appropriate. Secondary analyses will be considered exploratory, and no formal adjustment for multiplicity will be applied beyond the hierarchical testing of the coprimary end points.

The software R version 4.4.2 (2024-10-31 UCRT) has been selected to perform both design and data analysis.

### Feasibility Study

A feasibility study was conducted between September and October 2024. The purpose of the study was to evaluate the optimal timing for patient enrollment, the feasibility of data collection using REDCap and other digital tools, and the potential to recruit an adequate sample size. Initial feasibility results helped inform procedural aspects and adjustments to data collection tools.

### Ethical Considerations

This study will be conducted in accordance with the ethical standards of the responsible committee on human experimentation and with the World Medical Association Declaration of Helsinki. It will also adhere to the Convention for the Protection of Human Rights and Dignity of the Human Being with regard to the Application of Biology and Medicine [28]. Ethical approval will be granted by the Central-North-East Area Ethics Committee of the Veneto Region. The feasibility study has been approved by the institutional review boards of the University of Padova and Azienda ULSS 6 Euganea. Informed consent will be obtained from all participants prior to their inclusion in the study. Participants will be briefed on the nature, objectives, and potential consequences of the research. To ensure privacy and confidentiality, all personal data will be processed in strict compliance with the General Data Protection Regulation (GDPR) (EU) 2016/679. Data will be deidentified (pseudonymized) at the point of collection and stored on secure, encrypted servers accessible only to the authorized research team. No identifying information (such as names or hospital IDs) will be published. Participants will not receive any financial or nonfinancial compensation for their participation in this study.

## Results

In the feasibility study, 36 patients were screened, of whom 33 agreed to participate; 6 participants did not complete all questionnaire items. Initial technical issues related to the REDCap platform and the usability of digital devices were identified during the feasibility phase and were subsequently resolved.

The mean completion time was 7.67 minutes for the VLUK questionnaire and 5.12 minutes for the VeLUSET. VeLUSET scores indicated moderate-to-high confidence in general and daily self-care activities, with lower scores observed in domains related to ulcer management and trauma prevention. The feasibility results supported the acceptability of the questionnaires and informed minor procedural refinements without requiring changes to the study outcomes or measurement instruments.

The randomized controlled trial is currently pending approval by the local ethics committee. The study was funded in June 2025. Participant enrollment is expected to start in spring 2026, with an anticipated enrollment period of approximately 6 months and a total study duration of 18 months, including 12 months of follow-up.

At the time of manuscript submission, no participants had been enrolled in the randomized controlled trial beyond those included in the feasibility phase. Data analysis for the main trial had not yet been performed. The results of the randomized controlled trial are expected to be available and disseminated by the end of 2027.

## Discussion

Educational interventions represent a potentially efficacious method for promoting adherence to treatment programs and empowering patients with VLUs to assume an active role in the management of their disease. It can be suggested that patient education represents an effective strategy for improving the treatment of VLUs and reducing the incidence of relapse and the recovery time required.

Unfortunately, the time available for nurses to educate patients is often limited. This is due to the considerable number of patients and the necessity for an increasing number of nurses to manage the substantial workload. Therefore, it would be useful to use standardized educational methods to support the information given to the patient during treatment time. Such methods should be reliable, readily available, and allow the patient to access the information independently.

Although some educational interventions have been documented in the literature, it remains unclear which is the most effective [18].

A study compared a leaflet intervention with a video intervention and found that the video intervention was more effective in improving patients’ knowledge in this type of patient, although the results were not statistically significant [21]. Therefore, this study aims to improve the knowledge and self-care skills of patients with VLUs using a video-based educational intervention and to determine which intervention (video or brochure) is more effective.

Video interventions represent an accessible and cost-effective method of patient education, even for older adults. It is a low-cost approach that requires minimal time and resources, while providing nurses with the opportunity to educate patients in a convenient and effective manner. Moreover, it is easily available to users, thereby rendering it a highly accessible form of patient education. Additionally, an educational video provides access to accurate information from experienced professionals, even in the context of prevention or posthealing, when the patient is no longer required to attend outpatient nursing clinics for dressing.

This study has several notable strengths. Primarily, the use of videos in outpatient clinics allows nurses to prioritize personalized care. This is achieved by reducing the time spent on providing the same information to patients repeatedly and instead responding to their queries regarding content in the educational video that may be unclear to them. Moreover, as previously stated, the utilization of video technology could potentially reduce the costs associated with health care companies, thereby allowing for the reinvestment of these savings into other areas that may enhance the quality of service. Ultimately, the dissemination of educational videos is a relatively simple process, which could potentially reach individuals who are at risk of developing VLUs but have not yet sought medical attention for this condition. Consequently, the implementation of such videos could contribute to a reduction in the incidence of VLUs over time.

It is important to acknowledge that this study is not without limitations. For instance, the generalizability of the findings is restricted due to the fact that the study will involve only a single health care company. Nevertheless, should the study produce positive results, the educational intervention could be subjected to a replication study on a larger scale.

## Abbreviations

PUSH Tool 3.0: Pressure Ulcer Scale for Healing Tool 3.0
REDCap: Research Electronic Data Capture
SPIRIT: Standard Protocol Items: Recommendations for Interventional Trials
VeLUSET: Venous Leg Ulcer Self-Efficacy Tool
VLU: venous leg ulcer
VLUK: Venous Leg Ulcer Knowledge
WOW-I: Well-being of Wounds Inventory
